# Profiling Cognitive Reserve Among Community-Dwelling Older Adults: A Latent Class Analysis

**DOI:** 10.1093/geroni/igaf122.3830

**Published:** 2025-12-31

**Authors:** Wanrui Wei, Kairong Wang, Shuaifang Wei, Huan Zhang, Gabriella Engstrom, Azita Emami, Zheng Li

**Affiliations:** Yale University, Orange, Connecticut, United States; Peking Union Medical College Hospital, Beijing, Beijing, China (People’s Republic); Chinese Academy of Medical Sciences & Peking Union Medical College, Beijing, Beijing, China (People’s Republic); Chinese Academy of Medical Sciences & Peking Union Medical College, Beijing, Beijing, China (People’s Republic); Florida Atlantic University, Boca Raton, Florida, United States; Yale University, Orange, Connecticut, United States; Chinese Academy of Medical Sciences & Peking Union Medical College, Beijing, Beijing, China (People’s Republic)

## Abstract

Cognitive reserve (CR) is a multidimensional construct shaped by education, occupation, and leisure activities, yet traditional scoring approaches obscure heterogeneity among older adults. This study applied latent class analysis (LCA) to identify distinct CR profiles and examine their clinical correlates. A cross-sectional sample of 426 community-dwelling older adults aged ≥65 years in Beijing, China, was assessed using the Cognitive Reserve Index questionnaire (CRIq). Two CR profiles emerged: Class 1, characterized by low reading and leisure engagement (51.9%), and Class 2, defined by higher educational attainment and cognitively demanding occupations (48.1%). Compared with Class 1, Class 2 participants had higher Montreal Cognitive Assessment scores (26 vs. 25, p < 0.001), fewer subjective cognitive decline symptoms (3.5 vs. 4.5, p < 0.001), and lower frailty burden (Fried phenotype score: 0 vs. 1, p = 0.006). Class 2 membership was also associated with better chewing ability (p = 0.004), higher household income (p < 0.001), and greater likelihood of being male (p = 0.007). Age was inversely associated with Class 2 membership (β=–0.060, p = 0.024). These findings reveal meaningful heterogeneity in CR within aging populations and highlight the importance of stratifying older adults by CR profiles. Identifying subgroups at risk for cognitive decline and frailty provides new opportunities for targeted interventions to promote resilience for aging.

